# Biodegradable Nanoplastics: An Overlooked Polluting Terra Incognita Towards Global Plastic Risk Assessment?

**DOI:** 10.3390/nano16060371

**Published:** 2026-03-19

**Authors:** Xiaowei Wu, Shuai Tang, Kun Lu, Xiaoli Zhao

**Affiliations:** 1 Jiangsu Key Laboratory of Atmospheric Environment Monitoring and Pollution Control, School of Environmental Science and Engineering, Nanjing University of Information Science and Technology, Nanjing 210044, China; 003836@nuist.edu.cn; 2 Shanghai Key Laboratory for Urban Ecological Process and Eco-Restoration, School of Ecological and Environmental Sciences, East China Normal University, Shanghai 200241, China; 52213903021@stu.ecnu.edu.cn; 3 School of Environmental Science and Engineering, Zhejiang Gongshang University, Hangzhou 310018, China; 4 State Key Laboratory of Environmental Criteria and Risk Assessment, Chinese Research Academy of Environmental Sciences, Beijing 100012, China

**Keywords:** biodegradable plastics, nanoplastic pollution, biology, ecological risks, circular plastic economy

## Abstract

To mitigate environmental plastic accumulation and close the loop on plastic, the development of biodegradable plastics has presented a promising prospect for overcoming the global plastic pollution issue. However, it is critical to examine not only their benefits but also their unintended ecological consequences, especially for smaller-sized biodegradable nanoplastics. Our work highlights the often-overlooked risks associated with biodegradable nanoplastics. Due to the lack of environmental in situ monitoring data, the global occurrence, fate, and ecological risk of biodegradable nanoplastics remain poorly understood. Likewise, it remains unclear and questionable whether nanoplastics are eco-friendly as a promising alternative to the circular and sustainable plastic economy. We, therefore, call for a coordinated global effort to proactively mitigate the potential risks of biodegradable nanoplastics, including establishing a full-chain risk assessment system, developing key detection and simulation technologies, designing and optimizing bioplastic structures, and improving the legal supervision mechanism. These holistic efforts will facilitate the development of a sustainable practice for the closed-loop recycling of biodegradable plastics, which simultaneously helps establish a sustainable biodegradable plastic circular economy.

## 1. Introduction

To mitigate environmental plastic accumulation and close the loop on plastic, renewable feedstocks have been used to produce biodegradable plastics, including PHAs (polyhydroxyalkanoates), PLA (poly lacticacid), PBSA (poly(butylenesuccinate-co-adipate)), PBS (poly(butylenesuccinate)), and PBAT (poly(butyleneadipateterephthalate)) [1]. These offer promising prospects as substitutes for traditional nonbiodegradable plastic products, to alter the entire lifecycle of global plastics. It was estimated that global bioplastics production reached around 2.47 million tonnes in 2024, and is expected to increase to 5.73 million tons in 2029 [2]. Nevertheless, given the low rate of plastic recycling and upcycling, once biodegradable plastic replaces traditional fossil-based plastics (i.e., polyethylene (PE) and polypropylene (PP)), a significant amount of biodegradable plastic wastes will accumulate in the aquatic and terrestrial ecosystem after disposal. Emerging awareness suggests that, as human-made synthetic polymers, the uncontrolled use and production of biodegradable plastics simultaneously pose irreversible threats to global ecology, such as biodiversity, climate warming, and food security [3]. This is attributed to indiscriminately littered biodegradable plastic wastes undergoing biotic/abiotic weathering (mechanical cultivation, ultraviolet radiation, or biodegradation) during retention in the environment, leading to a reduction in molecular weight, alteration in surface roughness, and conversion of plastic films into smaller-sized nanoplastics (<1 μm in size) [4,5], possibly resulting in an alarming accumulation of micro(nano)plastics and chemical residues. Specifically, many studies have documented the aging and fragmentation of biodegradable plastics in the environment, resulting in the formation of large amounts of micro- and nanoplastics [6,7]. In addition, the aging rate of biodegradable plastics was reported to be higher than that of conventional plastics such as PE and PP. For example, Chen et al. exposed PLA and PP microplastics to water and UV irradiation for 24 h; the results showed that the size of PLA decreased by 47.7%, which is a larger decrease than that of PP microplastics (35.7%) [8]. Likewise, the photoaging rate of biodegradable plastic PBAT was also reported to be higher than that of PE, with the carbonyl index of aged PBAT (0.88) being >15 times higher than that of aged PE (0.06) during 10 days of ultraviolet exposure in soil [9]. The specific differences and similarities between typical/conventional and biodegradable plastics are listed in Table 1.

Therefore, a sobering issue is that continuous exposure to biodegradable nanoplastics could exacerbate environmental burdens on the ecosystem and human society triggered by particulate nanoplastics and organic chemicals during the entire plastic life cycle. It remains unclear whether biodegradable nanoplastics are truly eco-friendly and offer a promising alternative to the circular plastic economy over the long term. Before they are widely adopted, a full assessment exploring the ecological and evolutionary impacts of biodegradable nanoplastics on food security/human health is needed to reach a circular plastic economy and sustainable chemistry in plastic use.

## 2. Why Is Risk Assessment of Biodegradable Nanoplastics Becoming a Growing Need?

**Figure 1:**
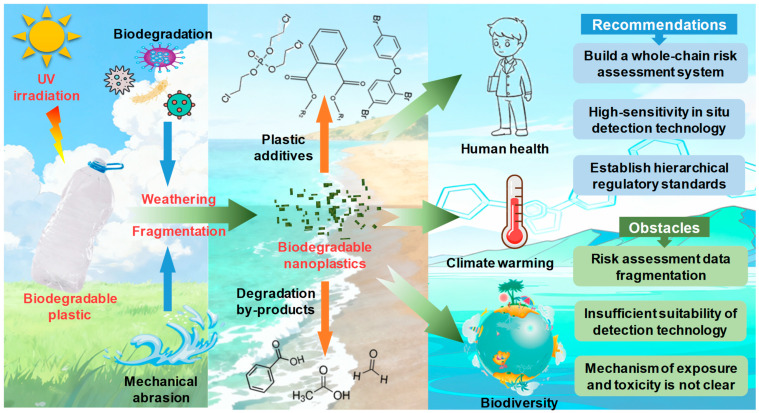
Formation and potential ecological risk of biodegradable nanoplastics in the environmental ecosystem.

After disposal, biodegradable nanoplastic particles are prone to inducing an irreversible threat to environmental ecology, such as soil biodiversity, climate warming, and food security (Figure 1) [3]. Some specific examples are as follows. (i) Biodiversity: For instance, accidental ingestion of biodegradable microplastics such as polylactate (PLA) by terrestrial organisms (earthworm: Eisenia fetida) could disrupt their intestinal digestive and absorptive functions [17]. After an earthworm encounters a pesticide (e.g., imidacloprid), PLA-imidacloprid co-exposure results in greater toxicity to the earthworm and higher earthworm mortality than PLA microplastic co-exposure does, owing to the PLA-imidacloprid mixture significantly interfering with the activities of superoxide dismutase (SOD), catalase (CAT), and acetylcholinesterase (AChE), resulting in negative effects on soil biodiversity through oxidative stress, DNA damage, and gene expression. (ii) Climate warming: Similarly to nonbiodegradable plastics like polyethylene (PE) and polypropylene (PP), biodegradable nanoplastics released into the environment are readily involved in microbial metabolic processes during long-term retention, affecting the subsequent carbon and nitrogen cycle in terrestrial and aquatic ecosystems, as well as greenhouse gas emissions, and further contributing to global warming [18,19,20]. (iii) Food security: Biodegradable nanoplastic accumulation in soils could hinder water infiltration, reduce water holding capacity, and further affect the growth of vegetables. Moreover, nanoplastic debris could be further enriched and absorbed by plants, reducing plant chlorophyll content, impairing plant photosynthesis ability, and further affecting global food security [21,22]. To address the effects of biodegradable micro(nano)plastics on agricultural ecology, a comprehensive analysis of the source appointment, environmental fate, and biogeochemical cycle in farmland is a prioritized issue.

Since biodegradable nanoplastic residue in the environment comprises polymer resin and additives (such as plasticizers and flame retardants), long-term mechanical activity and oxidation will simultaneously induce the release of chemicals, including plastic degradation products and additives, into the surrounding environment. These chemicals often pose serious health risks, and are readily able to exhibit unknown exposure risks to biodiversity, including genic, reproductive, or immune toxicity to organisms by interfering with cell mitochondrial metabolism (Figure 1) [4,23]. Specifically, a recent study reported that gastric lipase could mediate PLA microplastic (10 mg, 20 μm in diameter) fragmentation by 5% after 2 h of digestion in gastric lipase, simultaneously releasing 6.09 × 10^8^ particles/mL nanoplastics (200 nm in diameter) [4]. Correspondingly, gastric lipase-triggered degradation of PLA also readily released PLA oligomers with eight units (*m*/*z* 521.15), which can cause severe inflammatory effects via a decrease in the biological activity of immunomodulatory matrix metalloproteinase 12 (MMP12) and an increase in the concentration of anaphylatoxin (C3a, C5a, and TNF-α) [4]. These indicate that the widespread use of biodegradable plastic could pose a threat to ecology, rather than being completely harmless, which is often overlooked or misunderstood.

## 3. What Limitations Hinder Biodegradable Nanoplastics’ Risk Assessment?

To address the environmental risk of biodegradable nanoplastics, a comprehensive analysis of the source appointment, environmental fate and biogeochemical cycle is a prioritized issue. This section discusses the obstacles to environmental biodegradable nanoplastics risk assessment.

Firstly, due to the lack of standardized collection and analysis methods, long-term in situ monitoring experiments for the environmental concentration level, spatial distribution, and polymer composition of biodegradable nanoplastics are imperative [3]. This will be conducive to accurately quantifying the environmental occurrence, migration, and ecological threshold of environmental nanoplastics.

Secondly, current publications related to biodegradable nanoplastic pollution mainly focus on a single polymer type, particularly PLA [4,18], missing the toxicological data of other mainstream biodegradable plastics, including polybutylene adipate terephthalate (PBAT) and poly(butylene succinate) (PBS). These fragmented and isolated research data are not sufficient to systematically evaluate the bio-toxicity effects and ecological risks of biodegradable nanoplastics in the environment.

In addition, given that biodegradable nanoplastics can cause intestinal barrier damage and neurotoxicity, their underlying mechanism remains unclear. Specifically, in gut–brain axis-mediated nerve injury, the specific interaction chain between microbial metabolites and nerve signals is not yet clear; how the interference of oligomers on mitochondrial function causes genetic and immune toxicity still needs empirical support at the molecular level; there is almost no research on the cumulative effects of long-term low-dose exposure, which is precisely the key issue in the actual human exposure scenario; and the causal relationship between biodegradable nanoplastics exposure and diseases such as hyperuricemia and neurodegenerative diseases, especially the differences in susceptibility of sensitive populations including children and pregnant women, is still far from being understood. Based on the above considerations, before the widespread adoption of biodegradable plastics as a potential solution toward a circular economy, a full assessment exploring the ecological and evolutionary impacts of nanoplastics on food security/human health is needed to reach a circular plastic economy and sustainable chemistry in plastic use.

Moreover, quantification methodologies are an additional limitation for biodegradable nanoplastic risk assessment. Current research has already reported detection techniques for environmental micro- and nanoplastics, including optical photothermal infrared (O-PTIR), nanohyperspectral microscopic imaging, and NanoIR spectrometers [24,25], and these technologies have also been successfully applied to the transformation and environmental risk assessment of biodegradable nanoplastics in the environment [4,26]. However, in situ monitoring for the occurrence and concentration of nanoplastics, particular for biodegradable nanoplastics, in organisms is still limited, possibly due to the technical challenges that still need to be solved in the extraction and enrichment separation of environmental nanoplastics [27].

## 4. How Can We Overcome the Obstacles Related to Biodegradable Nanoplastics as a Polluting Terra Incognita?

To bridge these gaps, the scientific community should work together to achieve the following:

(i) **Establish a full-chain risk assessment system.** It is necessary to integrate environmental science and toxicological methods to build a nexus linking “material properties–environmental behavior–biological effects”, particularly through precise non-targeted screening methods, to accurately analyze the origins of plastic oligomers, clarify their transport and transformation patterns in soil–water–atmosphere systems, and evaluate toxicity variation mechanisms under climate change scenarios.

(ii) **Develop key detection and simulation technologies**. Developing high-sensitivity nanoscale particle detection technologies is key to achieving precise quantification and morphological analysis for biodegradable nanoplastics in environmental and biological samples. In vitro bionic models, such as a Caco-2 intestinal barrier model integrated with gut microbiota, should be established to simulate the in vivo metabolic processes of biodegradable nanoplastics [28]. Computational toxicology methods can be utilized to construct dose–response relationships, compensating for the limitations of animal testing.

(iii) **Design and optimize bioplastic structures.** By embedding engineered enzymes to accelerate biodegradation, we can thus prevent the release of toxic chemicals into the environment. For instance, Guicherd et al. embedded an optimized enzyme (protease from *Actinomadura*, PAM) (PLA depolymerase (PLAase), isolated from a thermophilic bacterium Actinomadura keratinilytica T16-1) to PLA matrix using a masterbatch-based melt extrusion process, which achieved full disintegration within 20–24 weeks under home-compost conditions [29]. In addition, replacing bio-based or biodegradable alternatives with toxic oligomers/additive residues in current bioplastics is also available to reduce the release of toxic chemicals into the environment.

(iv) **Improve the legal supervision mechanism.** The ecological risks and management tactics for biodegradable plastic and associated toxic chemicals should be addressed by a legally binding instrument (such as an international legally binding instrument on plastic pollution, www.unep.org/inc-plastic-pollution, 10 March 2026) to develop a comprehensive and effective international legal framework to curb the growing problem of plastic pollution and advance circular economies.

In summary, this Perspective highlights that, beyond conventional fossil-fueled nanoplastics, the environmental occurrence, fate, and risk of biodegradable nanoplastics are recommended to be considered as an overlooked yet important polluting terra incognita. More awareness is needed when utilizing biodegradable plastics as promising candidates to address global plastic pollution and overcome obstacles relevant to low-level exposure to toxic bioplastic oligomers (or additives). Additionally, the health risk assessment and waste management of biodegradable plastics should align with the requirements of fossil plastics as outlined in global treaties. These holistic efforts should be employed to establish a sustainable practice for the closed-loop recycling of biodegradable plastics, which simultaneously helps establish a sustainable practice for the biodegradable plastic circular economy.

## Figures and Tables

**Table 1:** Main differences and similarities between typical/conventional and biodegradable plastics.

Biodegradable Plastic	Conventional Plastic	Similarity	Differences	References
PLA	PE	Obvious cracks, holes and fragmentation can be produced on the surface of PLA and PE under UV irradiation.	PLA has a higher aging rate than PE. In addition, PE shows enhanced surface oxidation, while PLA is more reflected in the breaking of ester bonds.	[10]
PBAT	PE	Both PE and PBAT can undergo chain breakage, oxidation, and release oxygen-containing products and additives during photoaging in soil.	The photoaging rate of PBAT was significantly higher than that of PE.	[9]
PLA	PP	Both PLA and PP surfaces can be colonized by microorganisms and form biofilms, with the dominant phylum including Proteobacteria.	More bacteria with nitrification/denitrification function colonized on PP; lignin- and cellulose-degradation-associated bacteria readily colonized on PLA.	[11]
PLA	PP	Both of them can induce oxidative stress and cell membrane damage to earthworms in soil ecosystems.	PLA mainly relies on peroxidase to remove peroxides; PP relies more on glutathione-S-transferase.	[12]
PLA	PP	The surface of PP and PLA will change from smooth to cracks, wrinkles and holes during oxidation.	The surface of PLA contains more oxygen functional groups after aging, with its adsorption capacity to pollutants higher than that of PP.	[8]
PLA	PS	After aging, both PS and PLA have an increased adsorption capacity for tetracycline, and their surfaces will undergo aging as well.	The aging rate of PLA is higher than that of PS, showing more significant pitting, cracking, and fragmentation.	[13]
PLA	PVC	UV aging will alter the surface morphology, crystallinity, and surface charge of PLA and PVC, and consequently increase their adsorption capacity to antibiotics.	The adsorption capacity of PLA to antibiotics (NOR, CIP, and SMZ) is higher than that of PVC.	[14]
PLA	PE	Both PLA and PE could affect soil properties, microbial communities, and plant growth.	Under non-flooding and flooding conditions, the risk of PLA microplastics is higher than that of PE.	[15]
PLA, PBAT	LDPE	Both degradable plastics and refractory plastics can disturb the soybean rhizosphere microbial community and affect the soil element cycle.	Biodegradable microplastics are more likely to disturb soil rhizosphere microbial communities as well as soil element (e.g., carbon and nitrogen) cycle.	[16]

## Data Availability

No new data were created or analyzed in this study. Data sharing is not applicable to this article.
